# Effectiveness and safety profiling of zolpidem and acupressure in CKD associated pruritus: An interventional study

**DOI:** 10.1097/MD.0000000000025995

**Published:** 2021-05-28

**Authors:** Inayat Ur Rehman, Raheel Ahmed, Aziz Ur Rahman, David Bin Chia Wu, Syed Munib, Yasar Shah, Nisar Ahmad Khan, Ateeq Ur Rehman, Learn Han Lee, Kok Gan Chan, Tahir Mehmood Khan

**Affiliations:** a School of Pharmacy, Monash University, Jalan Lagoon Selatan, Bandar Sunway, Selangor Darul Ehsan, Malaysia; b Department of Pharmacy, Abdul Wali Khan University, Mardan, Khyber Pukhtoonkhwa; c Department of Nephrology, Institute of Kidney Diseases, Peshawar; d Department of Urology; e Department of Nephrology, North West General Hospital and Research Center, Peshawar; f Biomedical Engineering Technology, Foundation University Islamabad, Pakistan; g Novel Bacteria and Drug Discovery (NBDD) Research Group, Microbiome and Bioresource Research Strength, Jeffrey Cheah School of Medicine and Health Sciences, Monash University Malaysia, Bandar Sunway 47500, Selangor Darul Ehsan, Malaysia; h International Genome Centre, Jiangsu University, Zhenjiang, China; i ISB (Genetics), Faculty of Science, University of Malaya, Kuala Lumpur, Malaysia; j Institute of Pharmaceutical Sciences, University of Veterinary and Animal Science, Outfall Campus, Civil Lines, Lahore, Pakistan.

**Keywords:** CKD-associated pruritus, acupressure, zolpidem, hemodialysis, interventional study, quality of life, EQ5D-3L, PSQI, Urdu 5D-itch scale

## Abstract

**Background:**

Chronic kidney disease (CKD)-associated pruritus (CKD-aP) contributes to poor quality of life, including reduced sleep quality and poor sleep quality is a source of patient stress and is linked to lower health-related quality of life. This study aimed to investigate the effectiveness of zolpidem 10 mg and acupressure therapy on foot acupoints to improve the sleep quality and overall quality of life among hemodialysis patients suffering from CKD-aP.

**Method:**

A multicenter, prospective, randomized, parallel-design, open label interventional study to estimate the effectiveness of zolpidem (10 mg) oral tablets versus acupressure on sleep quality and quality of life in patients with CKD-aP on hemodialysis. A total of 58 hemodialysis patients having sleep disturbance due to CKD-aP completed the entire 8-week follow-up. The patients were divided into a control (acupressure) group of 28 patients and an intervention (zolpidem) group of 30 patients.

**Results:**

A total of 58 patients having CKD-aP and sleep disturbance were recruited. In the control group there was a reduction in the PSQI score with a mean ± SD from 12.28 ± 3.59 to 9.25 ± 3.99, while in the intervention group the reduction in PSQI score with a mean ± SD was from 14.73 ± 4.14 to 10.03 ± 4.04 from baseline to endpoint. However, the EQ5D index score and EQ-visual analogue scale (VAS) at baseline for the control group with a mean ± SD was 0.49 ± 0.30 and 50.17 ± 8.65, respectively, while for the intervention group the values were 0.62 ± 0.26 and 47.17 ± 5.82, respectively. The mean EQ5D index score in the control group improved from 0.49 ± 0.30 to 0.53 ± 0.30, but in the intervention group there was no statistical improvement in mean EQ5D index score from 0.62 ± 0.26 to 0.62 ± 0.27 from baseline to week 8. The EQ 5D improved in both groups and the EQ-VAS score was 2.67 points higher at week 8 as compared to baseline in the control group, while in the intervention group the score was 3.33 points higher at week 8 as compared to baseline. Comparing with baseline, the PSQI scores were significantly reduced after week 4 and week 8 (*P* =  < .001). Furthermore, at the end of the study, the PSQI scores were significantly higher in the control as compared to the intervention group (*P* = .012).

**Conclusion:**

An improvement in sleep quality and quality of life among CKD-aP patients on hemodialysis has been observed in both the control and intervention groups. Zolpidem and acupressure safety profiling showed no severe adverse effect other that drowsiness, nausea and daytime sleeping already reported in literature of zolpidem.

## 1 Introduction

Chronic kidney disease (CKD) has emerged as life-threatening problem globally^[1,2]^ and over past 10 years mortality due to CKD has increased by 31.7%.^[3]^ In Pakistan, every third person suffers from CKD,^[4]^ and there is an estimated annual increase of 100 per million population.^[5]^ Chronic kidney disease-associated pruritus (CKD-aP) is a bothersome complication experienced by 64% to 77.7% of patients on hemodialysis in Pakistan.^[6–10]^ CKD-aP contributes to poor quality of life, including reduced sleep quality.^[11–14]^ In Pakistan; 55% of patients have sleep disturbance due to CKD-aP and 13% of patients experience awakened states due to CKD-aP.^[10]^ Poor sleep quality is a source of patient stress and is linked to lower health-related quality of life.^[15]^

For CKD patients, the commonly used pharmacological treatments for sleep disturbance and insomnia are benzodiazepine receptor agonists of the non-benzodiazepine class, which are termed as “Z-drugs” (zolpidem, zaleplon, Zopiclone, and eszopiclone)^[16]^ benzodiazepine-receptor agonists (Temezapam, lorazepam, triazolam)^[16]^; orexin antagonist (Suvorexant)^[16]^ and melatonin (a pineal hormone).^[16–18]^ Among the pharmacotherapy for insomnia among CKD patients, in dialysis centers the nonbenzodiazepine hypnotics are an alternative due to no active metabolites, no physical dependence and few or no adverse events.^[19–21]^ No dose adjustment is required for zolpidem among CKD patients.^[16,17]^ A study on the effect of zolpidem for insomnia among hemodialysis patients reported no adverse effects such as amnesia and daytime drowsiness,^[22]^ and it improved sleep quality.^[22]^ Among nonpharmacological interventions for the treatment of insomnia, cognitive behavioral therapy (CBT),^[23]^ acupressure,^[23–30]^ physical exercise and a change of dialysis modality are widely used.^[23]^ Acupressure is a technique practiced in Traditional Chinese Medicine by stimulating acupoints, by applying pressure using the thumb, finger or hand. It is a noninvasive therapy that is linked to a low risk of side effects.^[31]^ Previous studies on acupressure among hemodialysis patients showed an improvement in sleep quality.^[32,33]^

Keeping in mind the prominence of impaired quality of life due to CKD-aP on hemodialysis, it is imperative to conduct an interventional study for improvement of sleep quality among patients with CKD-aP on hemodialysis. This randomized controlled trial compared the effectiveness of zolpidem versus acupressure in reducing the Sleep Quality Index (PSQI) score among CKD-aP patients on hemodialysis, and safety profiling both zolpidem and acupressure. The findings of this study will be helpful to clinical practice by giving future directions to treat sleep disturbance and improving the quality of life among CKD-aP patients on hemodialysis.

## 2 Materials and methods

**Figure 1:**
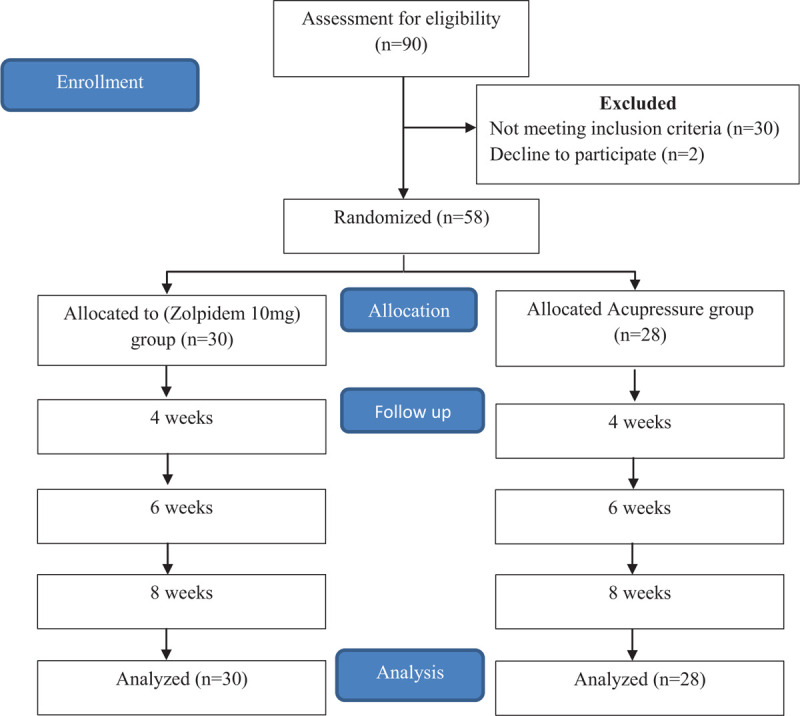
Study flow diagrams.

This was a multicenter, prospective, randomized, parallel-design, open label interventional study to estimate the effectiveness of zolpidem (10 mg) oral tablets versus acupressure on sleep quality and quality of life in CKD-aP patients on hemodialysis. The study was conducted at North West General Hospital & Research Center Peshawar, Pakistan and Institute of Kidney Diseases Peshawar, Pakistan. The study flow is presented in Figure 1.

### 2.1 Study duration and participants’ eligibility criteria

The duration of this study was from March 2018 to June 2018. The study comprised adult CKD-aP patients (above age of 18 years) on hemodialysis with affected sleep quality, and not on any medication to treat pruritus or sleep. The exclusion criteria was: patients having PSQI score less than 5.

### 2.2 Data collection and randomization of participants

The patient demographics, all subjective information and all laboratory parameters were recorded on a designed data collection form. The Urdu version of the 5D itch scale^[9]^ was used for assessment of CKD-aP, along with an Urdu version of the PSQI tool^[34]^ for eligibility of participants. Participants who had disturbed sleep and impaired quality of life due to CKD-aP were recruited.

Simple random sampling technique was used from a list of random numbers of eligible patients that was compiled by using the patient's hospital identification numbers. After recruitment the patients were requested to handpick an envelope from the basket indicating allocation to either intervention group or control group. Participants were randomized 1:1 into 2 groups, that is, a control group in which participants received daily acupressure therapy on acupoints KI-1(Yongquan) in both feet, and an intervention group that received zolpidem 10 mg tablets (orally) once daily. In the control group, the acupressure therapy was applied with an intensity adjusted to the patient's level of tolerance, for a total of 6 minutes (3 minutes per foot) per day, for 8 weeks. The sleep quality score on PSQI tool and quality of life score on EQ5D-3L was calculated at 3 and 2 different intervals respectively (i.e., baseline; week 4 and week 8) to measure the effect over time. However, at week 6 the participants in intervention group (zolpidem) were asked regarding any experienced side effects after taking the tablet once daily. Participants in the control group were questioned about acceptability toward acupressure on “Treatment Acceptability Questionnaire (TAQ).”

### 2.3 Safety assessment and severity of adverse drug reactions

**Naranjo algorithm** was used to measure recording and authenticating of drug-related events.^[35,36]^ The adverse event for zolpidem 10 mg tablets taken (orally) once daily were recorded; if any major adverse event had occurred, the therapy would have been considered for suspension. All the patients completing 6 weeks of zolpidem 10 mg oral tablets once daily were assessed for experienced adverse events.

### 2.4 Sample size

The sample size was based on a statistical superiority trial (continuous data) design of an interventional study. The hypothesis for this trial is that acupressure is a more effective therapy to improve sleep quality among CKD-aP patients, which has already been explained in the protocol published for this randomized controlled trial.^[37]^

### 2.5 Ethical considerations and data analysis

Informed written consent was taken from all participants before enrolling in this study. The study was approved by the Ethics and Research Committee of Institute of Kidney Diseases Peshawar, Pakistan; North West General Hospital & Research Center Peshawar, Pakistan and Monash University Human Research Ethics Committee. The trial protocol is registered under Australia New Zealand Clinical Trial Registry (Trial ID: ACTRN12618000001291). This study involving human participants is in accordance with the ethical standards of the institutional and/or national research committee and with the 1964 Helsinki declaration and its later amendments or comparable ethical standards. Statistical analysis was performed by using SPSS 22. Both the descriptive and inferential statistics were applied; participants’ demographic characteristics were expressed as frequencies (percentages) and mean ± SD for continuous normally distributed variables. As multiple measurements at different timelines were taken from each participant for sleep quality score using the PSQI tool, the general linear model is inappropriate. Generalized estimating equations (GEEs) are appropriate for analysis of longitudinal data and repeated measures.^[38]^ GEEs based on a correlation matrix AR (1) were used to estimate the improvement in PSQI score over time in individual patients, assessed on 3 occasions. Covariates (i.e., patient group, age, gender, marital status, history of renal disease, history of dialysis, and visits to dialysis center) were included to control for potential confounding effects. A Wald Chi-Squared test was used to test the significance of regression coefficients. The quality of life responses of participants were recorded on EQ-5D-3L questionnaire and the responses were coded to dichotomize the EQ-5D levels into “no problems” (i.e., level 1) and “problems” (i.e., levels 2 and 3).^[39]^ The EQ-5D-3L index scores were obtained by the crosswalk calculator methodology developed by Euro-QoL group.^[40,41]^ However, to compare baseline and week 8 of EQ5D index score and EQ-VAS, a pair *t* test was used. A *P* value of <.05 was considered statistically significant. Analysis was based on intention to treat.

## 3 Results

Upon the initial assessment, a total of 58 hemodialysis patients having sleep disturbance due to CKD-aP were eligible. The control (acupressure) group consisted of 28 patients, the majority (57.1%) of whom were males. The overall mean age of patients in this group was 44.42 ± 16.82 years (Mean ± SD). The intervention (zolpidem) group consisted of 30 patients, the majority (56.7%) of whom were male. The overall mean age of patients in this group was 44.80 ± 14.26 years (Mean ± SD). The majority (50%) of the patients in both control and intervention groups were illiterate. A majority of the patients (35.7%) in the control group had a history of chronic kidney disease for 3 to 4 years; while in the intervention group (40.0%) of patients had a history of chronic kidney disease for 1 to 2 years. However, 35.7% of patients in the control group and 30.0% of patients in the intervention group had a history of dialysis of less than 1 year. The majority of patients (78.6%) in the control group and 100.0% in the intervention group were doing dialysis twice a week (as shown in Table 1). Details of laboratory clinical value of participants at baseline are shown in Table 2, while details about the other common medications administered to the patients during the study period are given in Table 3.

**Table 1:** Demographics of respondents.

Statement	Total N = 58 (%)	Control group N = 28 (%)	Intervention group N = 30 (%)	*P* value
Gender				
* Male*	33 (56.9)	16 (57.1)	17 (56.7)	.971 a
* Female*	25 (43.1)	12 (42.9)	13 (43.3)	
**Age**				
* 18-30 years*	9 (15.5)	5 (17.9)	4 (13.3)	.281 b
* 31-40 years*	19 (32.8)	10 (35.7)	9 (30.0)	
* 41-50 years*	11 (19.0)	2 (7.1)	9 (30.0)	
* 51-60 years*	7 (12.1)	4 (14.3)	3 (10.0)	
* 61 years and above*	12 (20.7)	7 (25.0)	5 (16.7)	
* Mean age ±* *SD (years)*	44.81 ± 15.34	44.42 ± 16.82	44.80 ± 14.26	.815 c
Weight of patient (Kg)				
* Mean weight ±* *SD*	63.15 ± 13.38	65.21 ± 14.27	61.22 ± 12.43	.357 c
Education status				
* Illiterate*	29 (50.0)	10 (35.7)	19 (63.3)	.052 b
* Secondary level*	3 (5.2)	1 (3.6)	2 (6.7)	
* High school level*	17 (29.3)	9 (32.1)	8 (26.7)	
* Graduation*	2 (3.4)	2 (7.1)	0 (0)	
* Post-graduation*	7 (12.1)	6 (21.4)	1 (3.3)	
Marital status				
* Married*	47 (81.0)	21 (75.0)	26 (86.7)	.257 a
* Unmarried*	11 (19.0)	7 (25.0)	4 (13.3)	
History chronic kidney disease				
* < 1 year*	9 (15.5)	3 (10.7)	6 (20.0)	.104 b
* 1-2 years*	19 (32.8)	7 (25.0)	12 (40.0)	
* 3-4 years*	14 (24.1)	10 (35.7)	4 (13.3)	
* 5-6 years*	3 (5.2)	2 (7.1)	1 (3.3)	
* 7-8 years*	6 (10.3)	1 (3.6)	5 (16.7)	
* 9years and more*	7 (12.1)	5 (17.9)	2 (6.7)	
* Mean duration ±* *SD (years)*	2.98 ± 1.59	3.21 ± 1.59	2.77 ± 1.59	0.104 b
History of dialysis				
* < 1 year*	19 (32.8)	10 (35.7)	9 (30.0)	.429 b
* 1-2 years*	19 (32.8)	6 (21.4)	13 (43.3)	
* 3-4 years*	12 (20.7)	7 (25.0)	5 (16.7)	
* 5-6 years*	4 (6.9)	2 (7.1)	2 (6.7)	
* 7-8 years*	4 (6.9)	3 (10.7)	1 (3.3)	
* Mean duration ±* *SD (years)*	2.22 ± 1.38	2.36 ± 1.33	2.10 ± 1.02	.429 b
Dialysis frequency				
* Twice a week*	52 (89.7)	22 (78.6)	30 (100)	.008^∗^ b
* Thrice a week*	6 (10.3)	6 (21.4)	0 (0)	

a: Chi-Squared test; b:Fisher exact test; c:independent t test, ^∗^ statistically significant*P*vale <.05.

**Table 2:** Laboratory clinical values of participants at baseline.

Lab Tests	Control group	Intervention group
Uric acid	155 mg/dL ± 47.5	166 mg/dL ± 53.8
Serum Creatinine	893 μ mol/L ± 427.4	959 μ mol/L ± 423.3
Chloride	104 mmol/L ± 8.5	103 mmol/L ± 8.8
Potassium	5.1 mmol/L ± 0.7	5.2 mmol/L ± 0.8
Sodium	136 mmol/L ± 4.2	137 mmol/L ± 5.8
Magnesium	2.1 mg/dL ± 0.2	2.1 mg/dL ± 0.2
Phosphorous	4.9 mg/dL ± 1.1	4.7 mg/dL ± 0.9
Hb	10.3 g/dL ± 1.7	10.0 g/dL ± 1.0
WBC	6.9 × 103/cu mm ± 1.7	7.2 × 103/cu mm ± 1.6
Platelets	212 × 103 per mm^3^ ± 76.2	238 × 103 per mm^3^ ± 83.2

Value are shown in mean ± SD.

**Table 3:** Medications consumed by the patients.

Drugs	Strength	N
Nifedipine	30 mg	11
	60 mg	6
Amlodipine	5 mg	2
	10 mg	4
Furosemide	40 mg	6
	80 mg	4
Telmisartan	40 mg	1
Valsartan	80 mg	7
Losartan	25 mg	2
	50 mg	1
Bisoprolol	5 mg	6
Carvedilol	3.125 mg	1
	6.25 mg	5
Glimepiride	2 mg	1
Metformin	500 mg	1
Dipeptidyl peptidase-4 inhibitor	25 mg	1
	50 mg	1
Esomeprazole	20 mg	2
	40 mg	1
Omeprazole	20 mg	8
	40 mg	8
Simvastatin	10 mg	2
Atorvastatin	10 mg	1
Alfacalcidol	0.5 mcg	25
Sevelamer	400 mg	18
	800 mg	5
Erythropoietin	2000 iu	2
	4000 iu	12
Iron supplements		25
Ranitidine	150 mg	3
Aspirin	75 mg	3
Clopidogrel	75 mg	1
Xanthine oxidase inhibitor	80 mg	4

### 3.1 Assessment of CKD-aP and sleep score for eligibility of participants

The assessment of patients for eligibility criteria for having CKD-aP was carried out by Urdu 5D itch scale. Overall, the degree and direction were the 2 main domains affected by CKD-aP. However, disabilities in sleep (mean = 3.74 ± 1.26) and leisure/social activities (mean = 1.76 ± 1.11) were the 2 sub-domains mostly affected by CKD-aP. Overall, the mean score for the Urdu 5D itch scale was 10.88 ± 2.50 (range 7 to 19) as shown in Table 4. However, participants who had sleep quality score on the Urdu version of the Pittsburgh Sleep Quality Index (PSQI) of more than 5 (range 5 to 21) were eligible.

**Table 4:** CKD associated pruritus assessment for eligibility of patients on Urdu 5D itch scale.

Domain	Mean score ± Standard deviation
Duration	1.26 ± 0.84
Degree	2.26 ± 0.48
Direction	2.03 ± 0.72
Disability	
*Sleep*	3.74 ± 1.26
*Leisure/Social life*	1.76 ± 1.11
*House work*	1.40 ± 0.85
*Work/school*	1.38 ± 0.81
Distribution	1.55 ± 0.65
Mean 5D-IS score	10.88 ± 2.50
Range	7 to 19

### 3.2 Assessment of primary outcome:

#### 3.2.1 Comparison of PSQI score for control group and intervention group at baseline, week 4 and week 8

The PSQI score for baseline, week 4 and week 8 are shown in Table 5. At baseline, week 4 and week 8, the PSQI scores with a mean ± SD for the control group was 12.28 ± 3.59, 9.46 ± 3.90, and 9.25 ± 3.99, respectively. The PSQI scores with a mean ± SD for the intervention group at baseline, week 4 and week 8 were 14.73 ± 4.14, 10.13 ± 4.04, and 10.03 ± 3.89, respectively (as shown in Table 5).

**Table 5:** Mean score of Pittsburg Sleep Quality Index baseline and week 8 for control and intervention group.

	Control group			Intervention group		
PSQI score	N	Mean	S.D	N	Mean	S.D
Baseline	*28*	12.28	3.59	*30*	14.73	4.14
Week 4	*28*	9.46	3.90	*30*	10.13	4.04
Week 8	*28*	9.25	3.99	*30*	10.03	3.89

#### 3.2.2 Longitudinal assessment of the effect on PSQI score over time

Upon transforming the PSQI score (baseline, week 4 and week 8) into longitudinal data, GEEs were applied using PSQI as a dependent variable. By using GEE analysis to test the variance with group, the PSQI scores were significantly reduced after week 4 and week 8 (*P* =  < .001) in comparison to baseline. Furthermore, at the end of the study, the PSQI scores were significantly higher in the control as compared to the intervention group (*P* = .012). The interaction between timeline and patients group (control and intervention) was not significantly different but it can be observed that the PSQI score reduced in both patient groups (as shown in Table 6).

**Table 6:** Effect of other covariates on the PSQI score over time (n = 58).

Parameter	B	Wald Chi-Square	df	*P*-value	OR	95% CI
(Intercept)	12.209	175.616	1	<.001	200530.093	32960.302; 1220022.736
Dialysis duration						
* 3 years and more than 3 years*	0.737	0.519	1	.471	2.089	0.282; 15.496
* Less than 3 years*	0^a^				1	
Gender						
* Females*	−0.557	0.38	1	.538	0.573	0.097; 3.367
* Males*	0^a^				1	
Timelines						
* Week 8*	−3.036	31.833	1	**<.001**^**∗**^	0.048	0.017; 0.138
* Week 4*	−2.821	27.602	1	**<.001**^**∗**^	0.06	0.021; 0.171
* Baseline*	0^a^				1	
Patients groups						
* Control*	2.57	6.331	1	**.012**^**∗**^	13.06	1.765; 96.655
* Intervention*	0^a^				1	
Interactions						
* Week 8*^*∗*^* Control*	−1.664	3.43	1	.064	0.189	0.033; 1.102
* Week 8*^*∗*^* Intervention*	0^a^				1	
* Week 4*^*∗*^* Control*	−1.779	3.823	1	.051	0.169	0.028; 1.004
* Week 4*^*∗*^* Intervention*	0^a^				1	
* Baseline*^*∗*^* Control*	0^a^				1	
* Baseline*^*∗*^* Intervention*	0^a^				1	
(Scale)	15.516					

Dependent Variable: PSQI; Generalized linear model was used based on GEE, using working correlation matrix AR(1). a Set to zero because this parameter is redundant. ^∗^Significant; *P* < .05 was considered significant.

### 3.3 Assessment of secondary outcome:

#### 3.3.1 Quality of life assessment at baseline and end point

Upon transforming the responses of participants of EQ-5D levels into “no problems” (i.e., level 1) and “problems” (i.e., levels 2 and 3), a paired-samples *t* test was conducted to compare the EQ5D index score and EQ-VAS at baseline and week 8 for both control group and intervention group (as shown in Table 7). The results revealed that the mean EQ5D index score in the control group improved from a baseline score of 0.49 ± 0.30 to 0.53 ± 0.30 at week 8; while in the intervention group there was no statistical improvement in mean EQ5D index score from 0.62 ± 0.26 to 0.62 ± 0.27 (as shown in Table 7). However, regarding the mean EQ-VAS scores, in the control group there was a statistically significant improvement from a baseline score of 50.17 ± 8.65 to 52.85 ± 11.50 at week 8; while in the intervention group there was a statistically significant improvement from a baseline score of 47.17 ± 5.82 to 50.50 ± 9.31 at week 8 (shown in Table 7). On average the EQ-VAS score was 2.67 points higher at week 8 as compared to baseline in the control group, while in the intervention group it was 3.33 points higher at week 8 as compared to baseline, as shown in Table 7.

**Table 7:** EQ5D index score and VAS at baseline and week 8 for control and intervention group.

	Control group				Intervention group			
	Baseline (n = 28)	Week 8 (n = 28)	95% CI	*P* value	Baseline (n = 30)	Week 8 (n = 30)	95% CI	*P* value
EQ5D index score								
* Mean*	0.49	0.53	−0.091; 0.019	.187	0.62	0.62	−0.019; 0.017	.911
* SD*	0.3	0.3			0.26	0.27		
EQ VAS								
* Mean*	50.17	52.85	−4.686; −0.670	.011^∗^	47.16	50.5	−5.253; −1.413	.001^∗^
* SD*	8.65	11.5			5.82	9.31		

Paired t-test; SD: standard deviation; ^∗^*P* < .05 statistically significant.

### 3.4 Assessment of other outcomes

#### 3.4.1 Assessment of adverse drug reaction of Zolpidem and acceptability of acupressure

To ensure the participants’ safety in the intervention group for zolpidem 10 mg orally, drowsiness, nausea, and daytime sleeping were tested using Naranjo scale, as shown in Table 8. Acupressure therapy was highly acceptable among participants in the control group, as assessed by the “Treatment Acceptability Questionnaire (TAQ)” as shown in Table 9.

**Table 8:** Assessment for confirm adverse events for zolpidem (n = 30).

Adverse event	Confirmed (N)	Undecided (N)	Adverse events assessment based on Naranjo's algorithm
*Daytime drowsiness*	1		Probable
*Headache*		–	
*Amnesia*		–	
*Nausea*	1		Possible

**Table 9:** Individual scales of TAQ at week 6 of control group (n = 28).

Individual scales of TAQ	Mean	Std. Deviation
*Acceptability*	6.82	0.39
*Efficacy*	6.64	0.56
*Side effects*	1.00	0.00
*Trust rank of the therapist*	6.93	0.26

TAQ: treatment acceptability questionnaire. Acceptability: 1 = very unacceptable and 7 = very acceptable; Efficacy: 1 = “very ineffective” and 7 = “very effective”; Side effects: 1 = “very unlikely” and 7 = “very likely”; Trust rank of the therapist: 1 = “very untrustworthy” and 7 = “very trustworthy”.

## 4 Discussion

This interventional study was perhaps the novel of its kind to improve sleep quality among CKD-aP patients on hemodialysis by comparing both pharmacological (zolpidem) and nonpharmacological (acupressure) interventions for improvement in sleep at baseline, week 4 and week 8. In contrast, previous studies assessed the use of acupressure^[24–30]^ and zolpidem^[22,42]^ for the treatment of disturbed sleep among CKD patients on hemodialysis at one time. Sleep disturbance among CKD patients is often neglected by nephrologists, and no suitable option is available for the management of sleep disturbance, so CKD-aP contributes toward impaired quality of life among hemodialysis patients.

In our study, the assessment of patients in terms of the eligibility criteria for having CKD-aP was carried out by Urdu 5D itch scale. Overall, the degree and direction while in the disabilities sleep domain (mean = 3.74 ± 1.26) was mostly affected by CKD-aP, and the overall mean score for Urdu 5D itch scale was 10.88 ± 2.50. Similar findings were reported by a study done in Saudi Arabia on hemodialysis patients suffering from CKD-aP: duration, degree and direction while in disabilities sleep domain (mean = 3.30 ± 1.1) and leisure/social activities (mean = 2.90 ± 0.80) were affected by CKD-aP, and the overall mean score for pruritus was 19.1 ± 1.7.^[43]^

CKD-aP stimulates itching, and negatively affects sleep and quality of life.^[44]^ The itches are usually worse at nighttime and cause sleep disturbance.^[11,12,45–47]^ CKD-aP has been shown to be a cause of nocturnal awakenings and difficulty falling asleep.^[47,48]^ Pruritus patients have a compromised quality of life that is generally linked to limited personal freedom and control due to lengthy treatment time; overall, loss of freedom has wider implications, altering marital, family, and social relationships.^[49]^

In our study, the PSQI scores were taken at baseline, week 4 and week 8; both groups showed a reduction in the PSQI score, which is indicative of improvement in sleep quality. In the control group there was a reduction with a mean ± SD from 12.28 ± 3.59 to 9.25 ± 3.99; while for the intervention group there was a reduction in the PSQI score with a mean ± SD from 14.73 ± 4.14 to 10.03 ± 3.89 from baseline to week 8. Our results are aligned with another study in which there was a significant improvement in sleep score among hemodialysis patients at single point assessment by using acupressure therapy.^[24]^ A few studies have been done to study the effect of acupressure^[24–30]^ and zolpidem^[22,42]^ on sleep quality among hemodialysis patients, which showed improvement in sleep quality at a single point, but no study has been done among patients on hemodialysis suffering from sleep disturbance due to CKD-aP and at repeated time intervals, so we are unable to compare our results. Acupressure among hemodialysis patients led to an improvement in sleep.^[33]^ Similarly, findings about the effectiveness of acupressure in hemodialysis patients reported by another study revealed improved sleep quality due to acupressure in both the control and intervention groups.^[32]^ Acupressure results in a short-term improvement in sleep quality of hemodialysis patients and also brings comfort to patients, resulting in enhanced quality of life.^[28]^ In our study findings, patients in the intervention group receiving zolpidem 10 mg tablets daily reported improvement in sleep quality, as reflected by improvement in PSQI score, but the PSQI score remained above 5. This is similar to a study done in Iran that revealed that zolpidem (10 mg for patients younger than 60 years and 5 mg for older participants) significantly improves sleep quality in hemodialysis patients, though the PSQI scores remain above 5.^[22]^ Our study findings reveal that in comparison to baseline, the PSQI scores were significantly reduced after week 4 and week 8 (*P* =  < .001). Furthermore, at the end of the study the PSQI scores were significantly higher in the control as compared to the intervention group (*P* = .012). The interaction between timeline and patients’ group (control and intervention) was not significantly different, but it can be observed that the PSQI scores reduce in both patients’ groups.

In terms of quality of life improvement, our study findings reveal that the mean EQ5D index score in the control group improved from 0.49 ± 0.30 to 0.53 ± 0.30 at week 8; while in the intervention group there was no statistical improvement in mean EQ5D index score from 0.62 ± 0.26 to 0.62 ± 0.27. However, for the mean EQ-VAS score in the control group there was a statistically significant improvement from baseline score of 50.17 ± 8.65 to 52.85 ± 11.50 at week 8; while in the intervention group there was a statistically significant improvement from baseline score of 47.17 ± 5.82 to 50.50 ± 9.31 at week 8. On average, the EQ-VAS score was 2.67 points higher at week 8 as compared to baseline in the control group, while in the intervention group the score was 3.33 points higher at week 8 as compared to baseline.

In our study, acupressure was well tolerated and was highly acceptable, while 2 patients complained of side effects of zolpidem: daytime drowsiness and nausea, respectively. In the aforementioned study done in Iran looking at zolpidem to improve sleep quality in hemodialysis patients, none of the patients complained of any particular side effects.^[22]^

The age of patients recruited in both groups was not uniform which acted as a limitation on that part of the study; the patients were randomized 1:1 in both the control and intervention groups; also, some patients were receiving dialysis twice weekly and some were receiving thrice weekly. Furthermore, the pruritus score (Urdu 5D-IS) and PSQI was not of uniform but the score of CKD-aP of all patients was higher and was in the range of 5-25 which showed CKD-aP and for sleep disturbance the score was also in the range of 5-21 which confirm sleep disturbance.

## 5 Conclusion

Overall, improvement in sleep quality among CKD-aP patients on hemodialysis has been observed in both the control and intervention groups. An improvement in sleep quality and quality of life among CKD-aP patients on hemodialysis has been observed in both the control and intervention groups. Zolpidem and acupressure safety profiling showed no severe adverse effect other that drowsiness, nausea and daytime sleeping already reported in literature of zolpidem. Clinicians should consider providing acupressure therapy as an alternative method to improve the quality of sleep among CKD-aP patients on hemodialysis.

## 6 Strength and limitations

The strength of the study is that it is first trial that has been done to compare pharmacological and nonpharmacological intervention to improve sleep quality and quality of life among hemodialysis patient having CKD-aP. The limitation of study is that age of patients recruited in both groups was not uniform which acted as a limitation on that part of the study; the patients were randomized 1:1 in both the control and intervention groups.

## Author contributions

**Conceptualization:** Inayat ur Rehman, Learn Han Lee, Tahir Mehmood Khan.

**Data curation:** Inayat ur Rehman, Raheel Ahmed, Aziz Ur Rahman, Syed Munib.

**Formal analysis:** Inayat ur Rehman, David Bin-Chia Wu, Ateeq Ur Rehman.

**Funding acquisition:** Inayat ur Rehman, Kok Gan Chan.

**Investigation:** Inayat ur Rehman, Nisar Ahmad Khan, Tahir Mehmood Khan.

**Methodology:** Inayat ur Rehman, Raheel Ahmed, Ateeq Ur Rehman, Learn Han Lee, Tahir Mehmood Khan.

**Project administration:** Inayat ur Rehman, Ateeq Ur Rehman, Learn Han Lee, Kok Gan Chan, Tahir Mehmood Khan.

**Resources:** Inayat ur Rehman.

**Software:** Inayat ur Rehman.

**Supervision:** Aziz Ur Rahman, Nisar Ahmad Khan, Learn Han Lee, Tahir Mehmood Khan.

**Validation:** Inayat ur Rehman.

**Visualization:** Inayat ur Rehman, Syed Munib.

**Writing – original draft:** Inayat ur Rehman, Yasar Shah.

**Writing – review & editing:** David Bin-Chia Wu, Learn Han Lee, Kok Gan Chan, Tahir Mehmood Khan.

## Correction

When originally published, Peshawar was removed from affiliation c and has since been corrected.
