# Effectiveness and safety profiling of zolpidem and acupressure in CKD associated pruritus an interventional study: Erratum

**DOI:** 10.1097/MD.0000000000026345

**Published:** 2021-06-18

In the article, “Effectiveness and safety profiling of zolpidem and acupressure in CKD associated pruritus an interventional study”,^[1]^ which was published in Volume 100, Issue 21 of *Medicine*, a decimal was missing from Table 1 from the *Mean duration +/-SD (years)* column and *P* value row. The cell originally appeared as 0104 b and has been corrected to 0.104 b.
